# A workflow for semi‐automated volume correlative light microscopy and transmission electron tomography

**DOI:** 10.1111/jmi.13436

**Published:** 2025-06-30

**Authors:** Kohki Konishi, Guilherme Neves, Matthew Russell, Masafumi Mimura, Juan Burrone, Roland Fleck

**Affiliations:** ^1^ Nikon U.K. Branch of Nikon Europe B.V. Surrey UK; ^2^ Mathematical Science Research Laboratory Nikon Corporation Tokyo Japan; ^3^ Centre for Developmental Neurobiology Institute of Psychiatry Psychology & Neuroscience King's College London London UK; ^4^ Centre for Ultrastructural Imaging King's College London London UK; ^5^ Randall Centre for Cell & Molecular Biophysics King's College London London UK

**Keywords:** automated pipeline, correlative light and electron microscopy, electron tomography, image processing, microscopy sample navigation, synaptic ultrastructure

## Abstract

Volume Correlative Light and Electron Microscopy (vCLEM) is a powerful method for assessing the ultrastructure of molecularly defined subcellular domains. A central challenge in vCLEM has been the efficient navigation of Regions of Interest (ROIs) across multimodal and multiscale imaging datasets. We developed two key tools to overcome this challenge. First, we developed a multimodal image registration tool (SegReg) that utilizes segmentation of common objects across modalities and uses a Graphical Processing Unit (GPU) for registration of imaging datasets in two and three dimensions. Secondly, we developed a dedicated image viewer to visualize multimodal image registration in three dimensions (NavROI). Here, we demonstrate the integrated use of SegReg and NavROI to navigate large mouse tissue blocks with preserved fluorescent signals to allow selective targeting for TEM tomography of ROIs containing synapses and the cisternal organelle on the proximal region of the axon of a selected pyramidal neuron. By providing real time guidance to precise X‐Y trimming of selected ROIs, reliable estimates of cutting depth relative to ROIs and a clear visual navigation of multimodal and multiscale images, our integrated workflow significantly improves the efficiency and accessibility of vCLEM analysis.

## 1 INTRODUCTION

Electron microscopy (EM) has been a powerful tool for investigating the ultrastructure of cells and tissues. Volume EM (vEM), which enables three dimensional (3D) ultrastructural analysis, particularly for scales up to the tissue, organ, or even organism level, has gained significant attention.^1^, ^2^ Block‐face methods, such as serial block‐face scanning electron microscopy (SBF‐SEM)^3^ and focused ion beam scanning electron microscopy (FIB‐SEM),^4^ are widely used for acquiring vEM data by sequentially imaging the surface of a sample block. For example, SBF‐SEM has yielded novel insights into the development of synaptic ultrastructure.^5^, ^6^ Transmission electron microscopy (TEM) is also used for volume imaging in methods such as serial section transmission electron microscopy (ssTEM)^7^ and TEM tomography.^8^ In ssTEM, serial sections are collected on grids and imaged, and the volume from which the serial sections were taken is subsequently reconstructed. In TEM tomography, a thick section is imaged at a range of tilt angles and the section volume is tomographically reconstructed.^9^ ssTEM enables investigation of the dense network of axons, dendrites, and synapses that form neuronal circuits,^10^ whereas TEM tomography enables investigation of the details of biological ultrastructure with higher resolutions.^11^

vCLEM enables the 3D visualisation of the ultrastructure of fluorescently labelled targets and has unparalleled potential for assessing the ultrastructure of molecularly defined subcellular domains. Therefore, recent years have seen a surge in research on the development of vCLEM workflows to carry out vEM on fluorescently labelled targets.^12^, ^13^, ^14^, ^15^ One approach involves acquiring light microscopy (LM) images of fluorescently labelled cells, followed by resin embedding and vEM imaging using the ‘approach and correlation’ method.^16^ Another approach embeds the sample in resin in a way that preserves fluorophore activity, then iteratively performs LM imaging and stepwise trimming to navigate to fluorescently labelled cells for FIB‐SEM imaging.^17^, ^18^ A third approach incorporates X‐ray micro‐CT imaging between LM and vEM imaging.^19^, ^20^, ^21^, ^22^ However, navigation to TEM tomography has been limited to only a few researchers^16^, ^19^, ^20^ and is not widely used, largely because navigation to specific targets across microscopy modalities and spatial scales still remains massively labour‐intensive. This is particularly true for correlating LM images with TEM tomography.

Image registration is a crucial technique for navigation; therefore, several image registration methods have been proposed. One conventional method is for a user to manually identify landmark features that are visible in both images (e.g. cell nuclei) and transformation parameters of one image (e.g. rotation, translation, nonlinear warping) are calculated so that corresponding landmark features are matched when the image is overlaid onto the other. This method is implemented in tools such as Bigwarp^23^ and eC‐CLEM.^24^ While this method achieves high accuracy of image registration, manual landmark selection process is labour‐intensive. Consequently, automating image registration methods have been investigated. AutoCLEM was developed to automatically detect embedded beads and use them as landmarks for image warping.^25^ Intensity‐based image registration tools are freely provided as the Elastix toolbox^26^, ^27^ for medical image registration, and are implemented in a multi‐modal big image data sharing and exploration viewer (MoBIE).^28^, ^29^ Recently, CLEM‐Reg was developed to automatically establish correspondences between point clouds from two images.^30^ Developing an image viewer of multimodal image registration would provide an important tool to guarantee the navigation across modalities and spatial scales. Napari,^31^, ^32^ a multidimensional image viewer for Python, is a platform that is amenable to having applications programming interfaces developed for this purpose.

In this paper we present a novel integrated workflow for vCLEM that uses in‐resin fluorescence signals to allow semi‐automated navigation through millimetre‐sized resin embedded mouse brain tissue blocks and specific targeting of sub‐micrometre sized regions for TEM tomography. Our strategy starts with the creation of a reference map using LM encompassing the entire tissue block. ROIs are then imaged at higher resolution and mapped onto the reference map using newly created user‐friendly software that simplifies registering (SegReg) and displaying (NavROI) of imaging datasets acquired using different imaging modalities. Previously acquired LM images can be registered in real time with the video signal of the ultramicrotome camera to simplify the process of sample trimming in the X‐Y dimension. Further, we show that the fluorescence of ultrathin (200 nm) sections can be mapped with the reference map to produce reliable estimates of section Z depth. We used this approach to target TEM tomography to the proximal axonal region containing the Axon Initial Segment, where fluorescently labelled Gephyrin‐positive postsynaptic compartments and cisternal organelle are tagged with a specific fibronectin intrabody delivered using a viral expression approach.

## 2 RESULTS

### 2.1 Integrated imaging workflow

**FIGURE 1:**
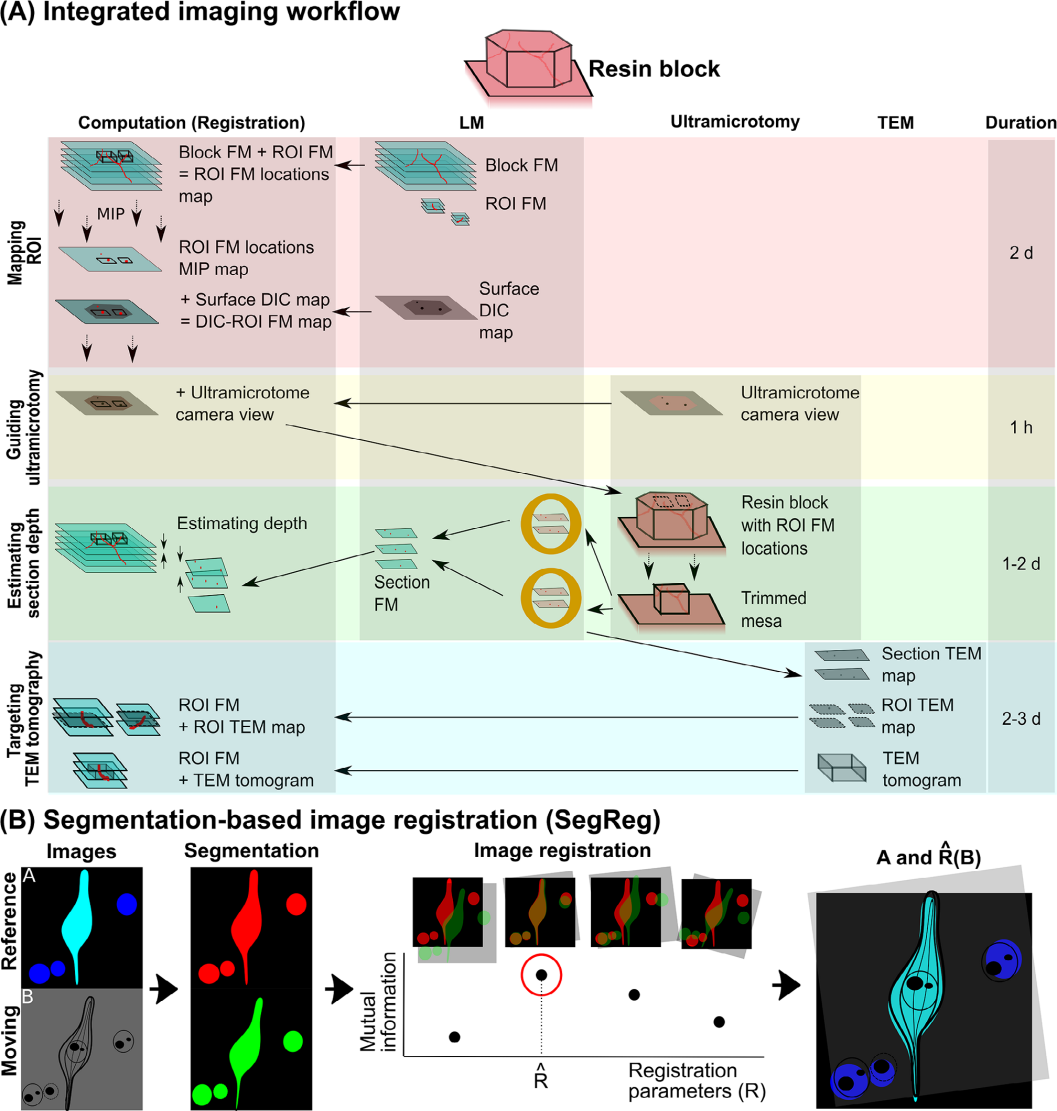
Integrated imaging workflow: RELATING (corRElative Lm And Tem mIcroscopy iNtegrated with Gpu‐based image registration). (A) Integrated imaging workflow which integrates imaging workflow and computation. Imaging consists of LM, ultramicrotomy, and TEM. The computation consists of two image registration algorithms: segmentation‐based image registration (SegReg), shown in B. The workflow comprises four steps: mapping ROI, guiding ultramicrotomy, estimating section depth and targeting TEM tomography. (B) SegReg algorithm. The algorithm consists of two primary components: segmentation and registration. The segmentation step segments common objects in the images from both modalities, either automatically or with user assistance. The registration step maximises a metric called mutual information (MI) while warping the moving image.

We developed an integrated workflow of navigating ROIs for volume CLEM, named as RELATING (corRElative Lm And Tem microscopy iNtegrated with Gpu‐based image registration). Figure 1A shows its overview. The workflow integrates imaging and computation across spatial scales, from LM imaging of sub‐millimetre scale to highlighting individual subcellular structures, up to the precise targeting of TEM tomogram imaging of ultrastructure. The computation consists of two multimodal image registration algorithms to register pairs of images in different modalities and/or scales, to facilitate navigation between them. The two algorithms are a novel segmentation‐based image registration (SegReg) algorithm shown in Figure 1B, which is described in the next section, and a conventional landmark‐based image registration algorithm.

The imaging workflow starts with a resin block containing brain tissue with fluorescently tagged markers (Figure 1A). We first acquired a fluorescence microscopy (FM) image stack of the entire resin block (Block FM, Figure 1A). The ROIs were visually identified and subsequently imaged at higher resolution (ROI FM, Figure 1A). To spatially register these image datasets, we generated a 3D map of the ROI locations within the entire block through SegReg (ROI FM locations map, Figure 1A). Since FM features within the block are not visible in the ultramicrotome stereomicroscope, a Differential Interference Contrast (DIC) image of the full surface of the resin block was also acquired at this point to relate the FM ROIs to the surface view in the ultramicrotome (Surface DIC map, Figure 1A). A maximum intensity projection (MIP) of the ROI FM locations map (ROI FM locations MIP map, Figure 1A) was then generated. Landmark‐based image registration was then employed to register the ROI FM locations MIP map with the Surface DIC map, essentially resulting in a projection of the lateral positions of the ROIs onto the surface view of the block (DIC‐ROI FM map, Figure 1A). We used this location map to identify the lateral position of ROIs when viewing the block surface in the ultramicrotome.

Next, the ultramicrotome camera view of the block was acquired and registered with the DIC‐ROI FM map through landmark‐based image registration. This process enabled the overlay of the projected lateral ROI locations onto the ultramicrotome camera view. By referencing this overlay during the trimming process, we optimised mesa trimming and reduced the danger of removal of tissue inside the ROIs.

We then proceeded with sectioning into the trimmed mesa to find sections at the depth of the ROI FMs. To find the sections at the correct depth, we collected sections onto cover slips and imaged using FM (Section FM, in Figure 1A). We defined the depth of each section as the depth corresponding to the optimal registration of SegReg between Section FM and Block FM. By this method we could estimate the depth of each section within the Block FM, and then relate this to the ROI FM locations. Thus, the sections whose estimated cutting depth matches the depth of the ROI FMs in the ROI FM locations map could be selected for subsequent correlative TEM imaging of those ROIs.

The final step involved targeting TEM tomography to specific ROIs identified in the high‐resolution ROI FM image stacks. TEM images of the whole of selected sections (Section TEM map, Figure 1A) were acquired. Within these images, specific ROIs were located and targeted for higher magnification TEM imaging (ROI TEM map, Figure 1A). SegReg was employed to register the ROI TEM map with the ROI FM map, enabling the overlay of target positions onto the ROI TEM map. TEM tomography was then performed at the target positions. Using SegReg again, the ROI FM was successfully registered with the TEM tomogram. The durations of each step are roughly 2 days, 1 h, 1–2 days and 2–3 days for mapping ROI, guiding ultramicrotomy, estimating section depth and targeting TEM tomography. If all the steps are performed without interruption, the average duration of this workflow is roughly 1 week.

### 2.2 SegReg for multimodal images

We developed a SegReg algorithm for registering pairs of multimodal images acquired from different microscopy modalities and/or scales. Figure 1B presents a flowchart illustrating the algorithm. One image serves as the reference image (Image A in Figure 1B), while the other serves as the moving image (Image B in Figure 1B). The algorithm primarily consists of two primary components: object segmentation and image registration.

For object segmentation, we employed automated or user‐assisted techniques to segment objects that are frequently present in both images. Segmentation masks were subsequently converted into labels via connected component labelling. Examples of such common objects include blood vessels, cell nuclei, and neurons.

For image registration, we employed a method to perform registration using the mutual information (MI) metric. The image registration works by calculating transformation parameters that register the moving image with the reference image, maximising the MI value between the two.^33^ In practice, the method involves warping the moving image, constructing a joint intensity histogram, and calculating the entropy of both individual images as well as their joint entropy. MI is defined as the difference between the sum of the individual image entropies and the joint entropy. By maximising the MI value, the method identifies the optimal affine transformation parameters for image registration. We randomly generated affine transformation parameters, warped the moving image, and computed the MI values between the labels from images A and B in the Fourier domain using a GPU.^34^

#### 2.2.1 NavROI for ROI navigation

To streamline the integrated workflow, we developed a ROI navigation viewer, NavROI, using napari that displays the spatial relationships between multiple images (Figure S1). The images can be multiple images and image stacks in two or three dimensions. NavROI accepts a configuration file consisting of the paths of the images and their registration parameters, which is an output from SegReg (‘segreg’) or conventional landmark‐based image registration method (‘landmark’). NavROI also accepts a Z magnification factor compared to X and Y, a registration method (‘segreg’ or ‘landmark’), and tick box whether image boundaries are displayed. NavROI visualises locations of each image relative to the image with the largest field‐of‐view.

### 2.3 Implementation of the integrated workflow to mouse brain tissue

We applied the integrated workflow to a resin block in which mouse brain tissue was embedded. The primary targets are their synapses and the cisternal organelle. By employing correlative imaging at the level of TEM tomography, the quantitative relationship between ultrastructural features – such as vesicle numbers – and functional properties indicated by fluorescence intensity can be investigated. This investigation will be essential for developmental neurobiology and neuropsychiatric drug discovery.

#### 2.3.1 Mapping the ROI FM to the Block FM

**FIGURE 2:**
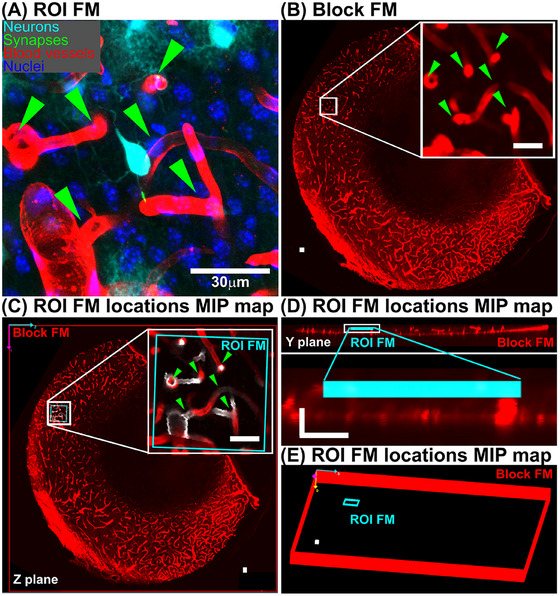
Mapping the ROI FM within the Block FM. (A) ROI FM of mouse brain tissue: Neurons in cyan, synapses in green, blood vessels in red, and nuclei in blue are visualised. Five structures in the blood vessel channel are indicated by green arrowheads. (B) Block FM blood vessel channel: a volume rendering highlights the region containing the ROI FM, white box. A zoomed‐in view is shown in the inset, with five structures corresponding to those in panel a marked by green arrowheads. All the size of white scale bars and cubes in this Figure are 30 µm. (C–E) ROI FM Location Maps: (C) NavROI *Z*‐plane view: a screenshot of the NavROI in *Z*‐plane view. Blood vessels from ROI FM are shown in white. (D) NavROI *Y*‐plane view: screenshots of NavROI in *Y*‐plane view, showing both the entire view, top panel, and a zoomed‐in region around the ROI FM, bottom panel. The size of the L is 20 microns. (E) Spatial relationship: a NavROI screenshot illustrating the spatial relationship between the ROI FM and the Block FM.

Figure 2A shows an example of ROI FMs of mouse brain tissue. Neurons are shown in cyan, synapses in green, blood vessels in red, and nuclei in blue. The neuron centred in the ROI FM is surrounded by distinctive blood vessels. Selected distinctive regions of the blood vessel structures are indicated by green arrowheads. Since the blood vessel structure is heterogeneous and shows good coverage across the tissue, we used the blood vessel channel to map the ROI FM locations to the Block FM. The region containing the ROI FM is highlighted in the white box and its volume rendering is shown in the inset. The five green arrowheads in Figure 2B are the corresponding points in Figure 2A.

To map the ROI FM to the Block FM, we utilised image registration and visualisation techniques. SegReg was employed to determine the optimal registration parameters between these two image stacks. Subsequently, we utilised NavROI for visualisation by inputting the file paths of the ROI FM, Block FM, and the optimal registration parameters.

Figure 2C and D presents screenshots of the NavROI interface. Figure 2C highlights its *Z*‐plane viewer, where the blood channel images from both ROI FM and Block FM are superimposed. The ROI FM region is highlighted with a cyan‐colored box, the ROI FM is visualised in greyscale, and the Block FM in red. A zoomed‐in view with the guiding arrowheads is shown in the lower right corner. Figure 2D illustrates the *Y*‐plane view of the NavROI, with the upper panel showing an overall view and the lower panel showing a zoomed‐in view of the white box around the ROI FM.

Figure 2E depicts a screenshot of a bird's‐eye view generated by NavROI, enabling visualisation of the mapping the ROI FM region in cyan to the Block FM region in red. This visualisation is utilised for lateral trimming of blocks and section selection, as detailed below.

#### 2.3.2 Guiding the lateral trimming of the block during ultramicrotomy

**FIGURE 3:**
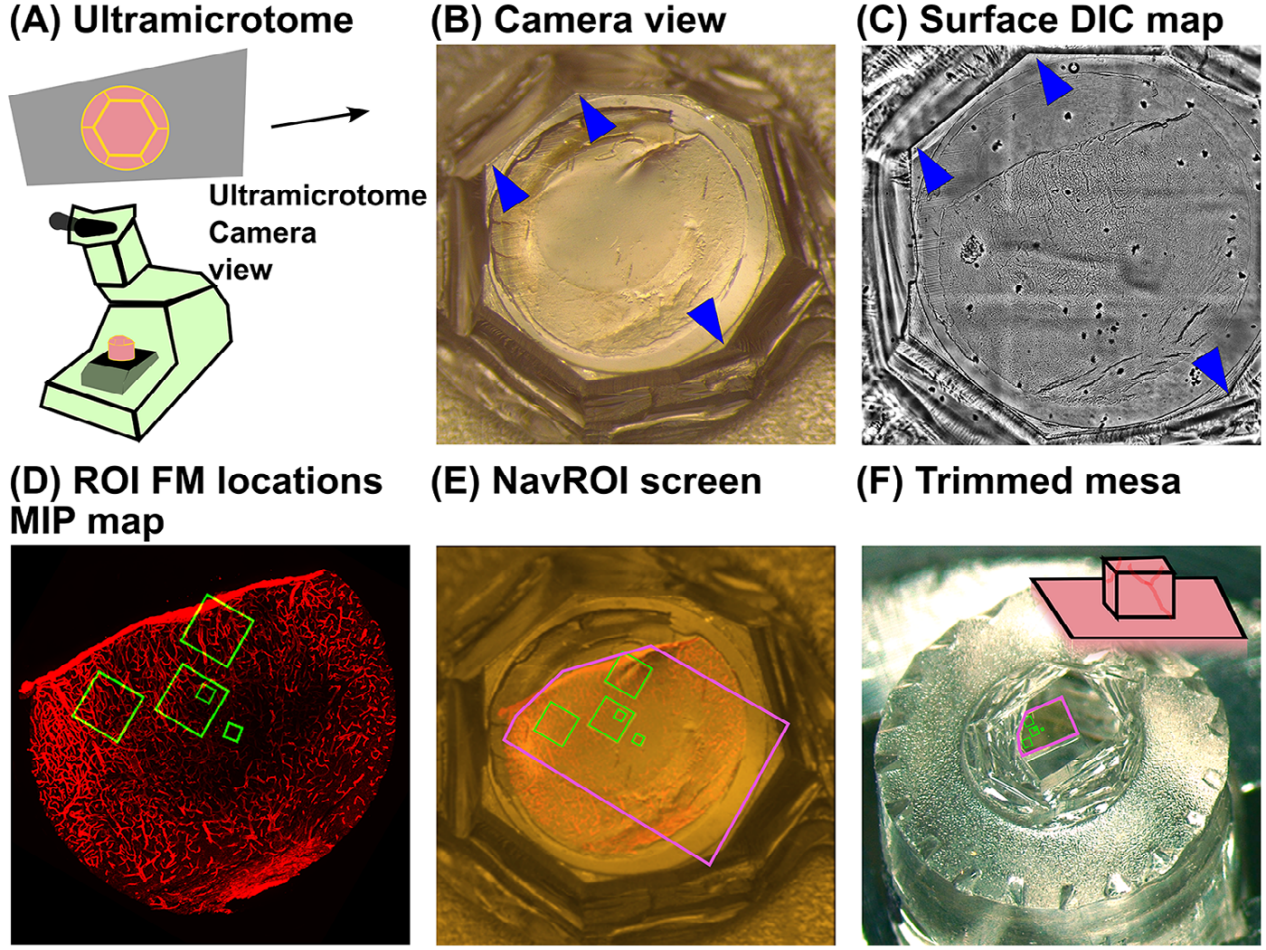
Using a surface Differential Interference Contrast (DIC) map of the block to guide ultramicrotomy. (A) A schematic of the UC and its camera view on top. (B) An UC camera view of the block. Three landmarks of the block corners are indicated by blue arrowheads. (C) A surface DIC map of the block. The landmarks corresponding to those in B are indicated by blue arrowheads. (D) A ROI FM locations MIP map. Five regions of ROI FMs are shown in green. (E) A screen shot of NavROI illustrating an UC camera view overlaid with five ROI FMs. The trimming region is shown in pink. The Surface DIC map and the ROI FM locations MIP map are also shown. (F) An UC camera view of the trimmed block. The trimmed region is shown in pink.

We optimised trimming of the block mesa by overlaying ROI FM locations onto the ultramicrotome (UC) view of the block. Figure 3A illustrates a schematic of the UC system, with the camera view displayed on the top screen. Figure 3B presents a UC camera view of the block before trimming. Three landmarks of the block corners are marked with blue arrowheads. Figure 3C shows a Surface DIC map of the block. The landmarks corresponding to those in Figure 3B are indicated by blue arrowheads. Figure 3D displays a ROI locations MIP map. The five ROI locations are highlighted as green rectangles. Since the surface DIC map is registered with the ROI FM locations MIP map during acquisition, we obtained an overlay of ROI locations on the UC camera view. Figure 3E shows the overlay where the planned trimming region enclosing all the ROIs is highlighted in pink. We trimmed away materials outside of the pink polygonal region using this overlay as a guide. Figure 3F shows a UC camera view of the trimmed block.

#### 2.3.3 Estimating cutting depth of sections

**FIGURE 4:**
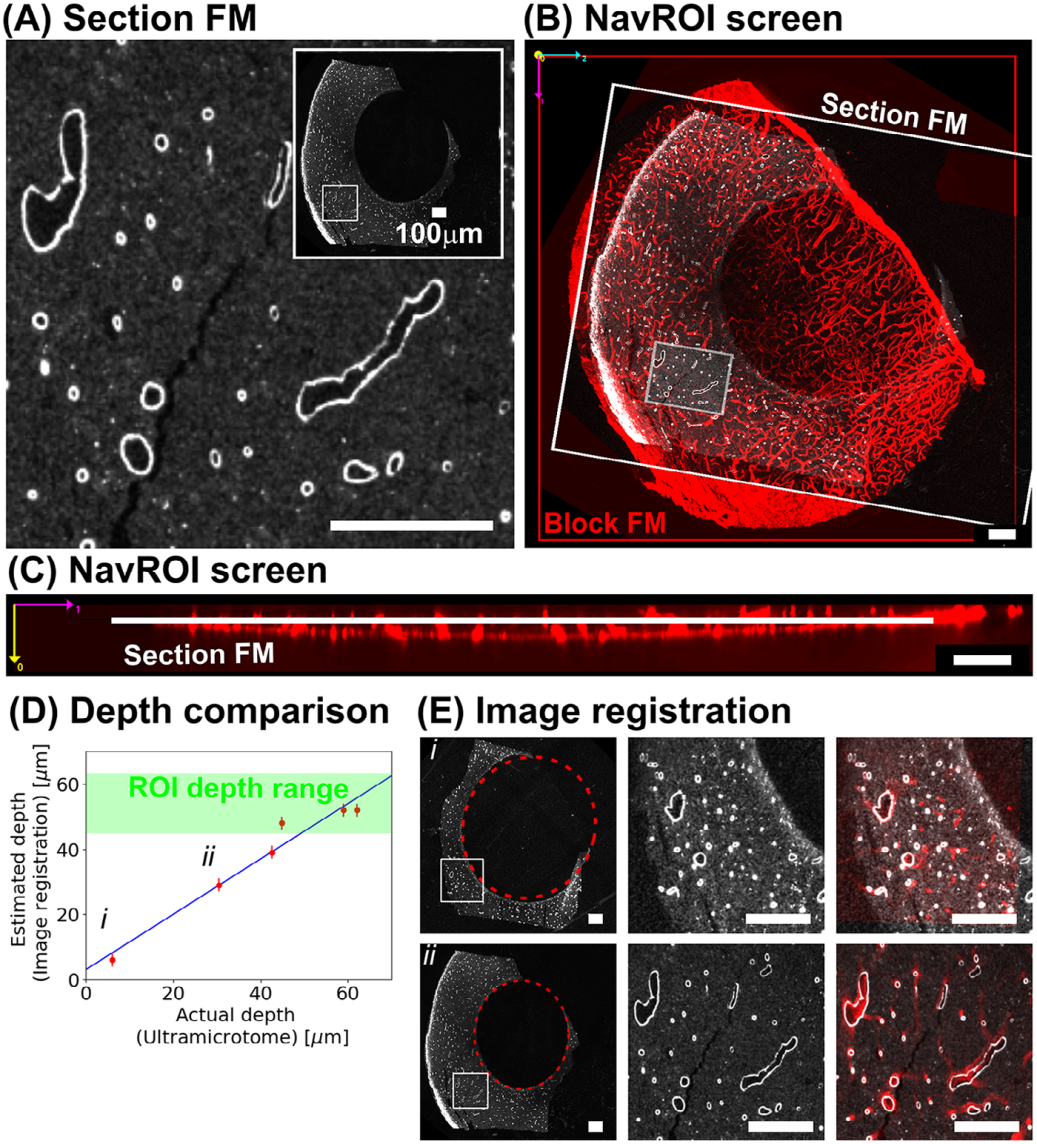
Estimating cutting depth of sections. (A) A local region of the blood vessel channel image of a Section FM. Its entire image is shown in the inset. All of the scale bars in this Figure are 100 µm. (B, C) A Section FM is displayed relative to the Block FM using NavROI: (B) a screen shot of NavROI in Z plane view and (C) a screen shot of NavROI in Y plane view. (D) Comparison of the feeding values from the UC and the depth estimation using image registration. The details of depth estimation for the leftmost point *i* and point *ii* to its right in the plot are shown in E. (E) The image registration for depth estimation. Left: An entire section image with a white rectangle. The white rectangle region is used for depth estimation. The inside the dotted red circle shows an empty resin region. Middle: The zoomed‐in view of the white rectangle region. Right: The result of image registration for depth estimation. The top row: point *i*, the bottom row: point *ii*.

We estimated cutting depth of sections by reading the depth from the Block FM surface at the point of optimal image registration between Section FM and Block FM through SegReg. Figure 4A shows a local region of blood vessel channel image of Section FM, with the full section shown in the inset. We focused image registration analysis to the local region of a few hundred‐micron size to mitigate nonlinear distortions in the Section FM. Subsequently, we utilised NavROI for the mapping process by inputting the file paths of the entire Section FM, its local region, Block FM, and the optimal registration parameters.

Figure 4B and C presents screenshots of the NavROI interface. Figure 4B displays its *Z*‐plane view, where the Section FM and its local region are highlighted with white boxes, and the blood vessel channel of the Block FM is visualised in red. Figure 4C illustrates the *Y*‐plane view of the NavROI. This view visualises the depth of the Section FM from the surface of the Block FM.

Figure 4D compares the cutting depths of the six sections relative to the block surface with the corresponding feeding values from the UC. Section FMs were used to generate first two points nearest to the block surface and Section TEM maps were used to generate the remaining points. The green region indicates the depth range estimated from registering the ROI FM in the Block FM. A diagonal line with a slope of 1 is also plotted for reference. Linear regression analysis of the plot yielded a slope of 0.85 and an intercept of 3.00 µm. The first two sections closest to the block surface are labelled *i* and *ii* in the plot.

Figure 4E provides detailed process of the image registration for the Section FM labelled *i* and *ii*. The left panel displays the entire Section FM, with the local region used for registration highlighted in white rectangles. Note that the centre of the Section FM contains no resin, as indicated by the dotted red circles. The middle panel shows a zoomed‐in view of the local region in the left panel. This local region was utilised for image registration. The right panel presents the optimal image registration result between the Section FM and the Block FM. The section image is depicted in grey, while the corresponding region of the Block FM is shown in red.

#### 2.3.4 Targeting TEM tomography to specific ROIs in FM images

In the previous sections, we utilised blood vessels in navigating ROIs. Here, we show navigation from the FM ROI to targeted TEM tomography of targets along an axon identified within the FM ROI using nuclei and neurons.

**FIGURE 5:**
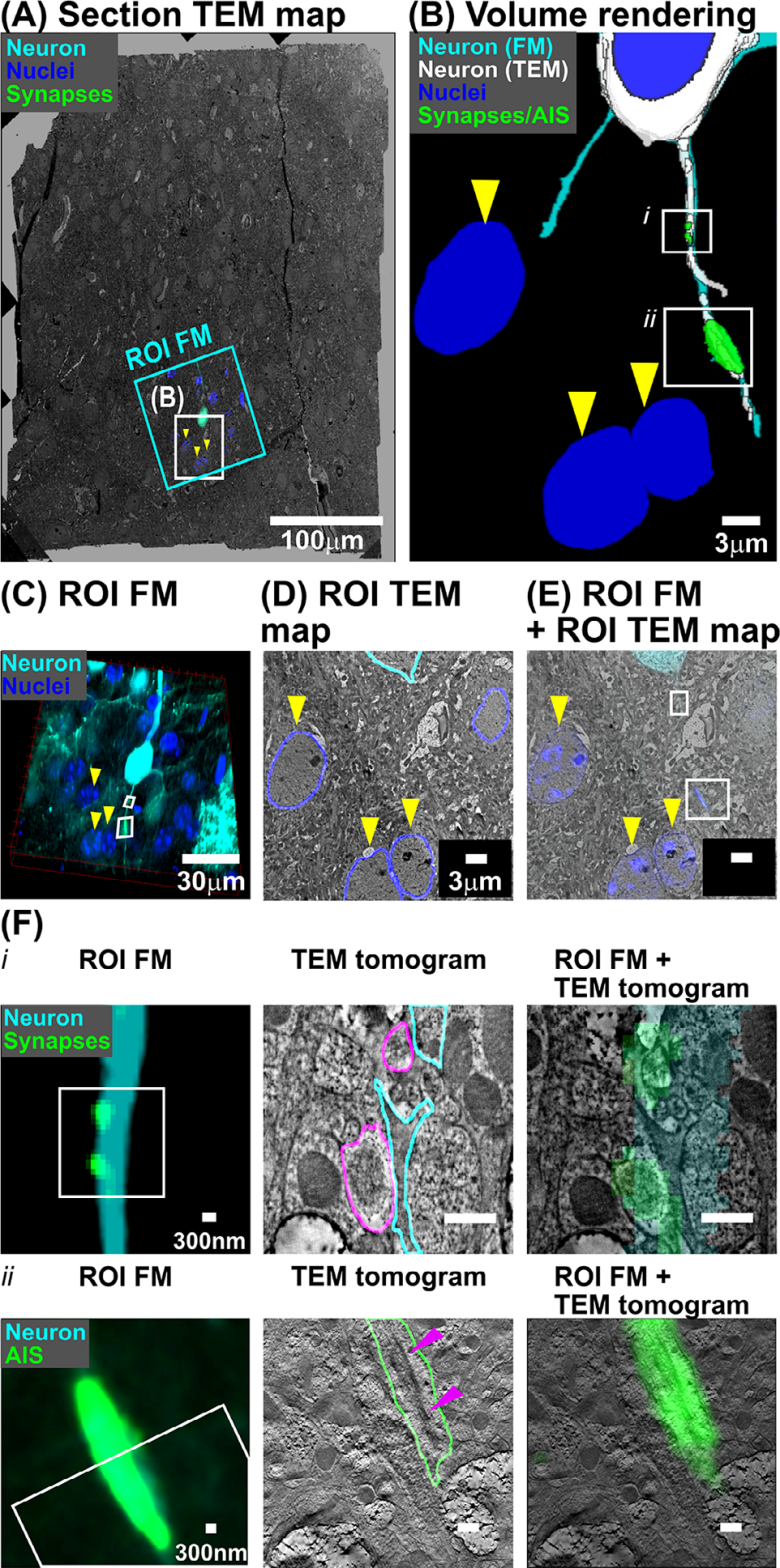
Targeting TEM tomography to synapses identified in confocal images. (A) An overlay of a ROI FM on a Section TEM map. The region surrounded by a white rectangle contains an axon of a neuron and is magnified on the right panel. Surrounding nuclei are indicated by yellow arrowheads. (B) A volume rendering of the segmentations of the neuron from the ROI FM and the ROI TEM map, displayed in cyan and white. The two target regions for TEM tomography are shown in green and highlighted in a white rectangle. Surrounding nuclei are shown in blue. (C) A 3D view of the ROI FM. (D) An example of the ROI TEM map with segmentation. (E) The overlay of the ROI FM on the ROI TEM map. (F) Image registration at the target regions. Left: The target region in the ROI FM, the cytoplasmic marker channel in cyan and synapse channel in green. The corresponding region to the Middle panel is shown in white rectangle. Middle: The TEM tomogram around the region with segmentation. Right: Their overlay. The top row: *i*. Synapses in the axon. Presynaptic boutons, outlined in purple, correspond to postsynaptic densities identified by fluorescent labelling. The bottom row: *ii*. An accumulation of postsynaptic scaffold protein likely representing cisternal organelle, indicated by a purple arrowhead, is a distinctive feature expected to be found in the AIS.

Figure 5A presents a Section TEM map with an overlaid ROI FM. The ROI FM is marked by a cyan box. A neuron is shown in cyan, and nuclei in blue. A white box emphasises a region containing the axon, which is encircled by three nuclei indicated by yellow arrowheads.

Figure 5B presents a volume rendering of the axon segmented from the ROI FM and the ROI TEM map, shown in cyan and white, respectively. Segmentation of synapses along the axon of the neuron and an accumulation of postsynaptic scaffold protein likely representing cisternal organelle from the ROI FM are shown in green. These are the TEM tomography targets and are further emphasised by white rectangles labelled *i* or *ii*. Additionally, nuclei segmentation from the ROI FM is rendered in blue with yellow arrowheads.

To reconstruct the axon from serial ROI TEM maps, these maps should be registered. We registered each ROI TEM map to the FM Block using SegReg algorithm. Figure 5C presents a volume rendering of the FM Block, highlighting neurons and nuclei in cyan and blue. The two target regions are outlined by white rectangles. Figure 5D shows an example ROI TEM map, overlaying neuron and nuclei segmentations in cyan and blue. Figure 5E demonstrates optimal image registration between Figure 5C and D. By examining the overlay of fluorescent signals on the ROI TEM map, Figure 5E, we segmented the axon from the ROI TEM map. Repeating this registration process for all ROI TEM maps achieved axon segmentation and subsequent reconstruction.

To identify targets in ROI TEM maps, we performed nonlinear image registration between adjacent ROI TEM maps by focusing on the local region of a few 10‐µm size containing targets. This additional step was necessary because the initial linear image registration was not sufficient to trace the axon across adjacent ROI TEM maps due to residual nonlinear registration errors. By employing nonlinear image registration, we overlaid the fluorescent signal of targets onto the ROI TEM maps. After nonlinear image registration, we proceeded to acquire TEM tomograms.

We registered the FM Block to TEM tomograms through SegReg as illustrated in Figure 5F. Zoomed‐in regions of ROI FM are shown in the left panel, alongside corresponding TEM tomograms with segmentation overlays, the middle panel, and optimal image registrations, the right panel. Upper panels depict the synapses in the axon, while lower panels show an accumulation of postsynaptic scaffold protein likely representing cisternal organelle.^35^

## 3 DISCUSSION

### 3.1 Efficient navigation of ROIs using integrated vCLEM workflow

vCLEM offers unparalleled potential for assessing the ultrastructure of molecularly defined subcellular domains. Image registration techniques between light and electron microscopy images have been developed by several authors^23^, ^24^ and a central challenge in vCLEM has been the efficient navigation of ROIs across multimodal and multiscale images. Here we report an integrated vCLEM workflow with SegReg as well as NavROI from confocal microscopy images of the tissue block to high‐resolution TEM tomography of the tissue sections.

### 3.2 Robust, real‐time ROI navigation with SegReg

Robust and real‐time image registration algorithms are crucial for ROI navigation. Conventional methods demand manual landmark correspondence between reference and moving images, a time‐consuming task. To streamline this process, automated registration methods have been developed.^25^, ^30^, ^36^ However, these methods often assume that both images are acquired from the same region, which may not be feasible in scenarios like serial section high‐resolution TEM imaging.

Our novel image registration algorithm addresses this limitation by enabling immediate registration after acquiring a single image of a section, eliminating the need to wait for the entire series. By leveraging GPU acceleration for efficient computation of MI, we achieve real‐time performance. This allows for daytime imaging and same‐night registration, providing timely feedback for informed decision‐making. The next morning, users can decide whether to proceed with the planned imaging depth or adjust the number of sections to reach the desired ROI. This real‐time capability enhances the robustness and efficiency of ROI selection. While we employed segmentation for image registration, generative adversarial network‐based approaches^37^ might also be applied to multi‐modal image stack registration.

Multi‐modal image registration algorithms have been extensively studied in medical imaging. For images where intensity values are positively correlated, such as MRI and CT images, MI has proven effective.^38^, ^39^ Segmentation‐based approaches have been proposed for registering T1 and T2 MRI images.^40^ Algorithms for image stack registration have also been actively explored.^41^

Inspired by advancements in medical imaging, we propose a SegReg method for vCLEM. Given the extensive research on segmenting LM and TEM images,^42^ our method leverages these developments. We focused on segmenting key organelles: blood vessels, cell nuclei, and neurons. Blood vessel segmentation can be accomplished using either simple thresholding or machine learning techniques. For cell nuclei, we employed SAM, but Cellpose 2^43^ can be an option. Neuron segmentation can benefit from machine learning technique.^44^, ^45^, ^46^

In order to decide the image registration metric of the SegReg, we initially compiled a list of metrics for multimodal image registration from the literature. These metrics included MI,^33^ cross entropy,^47^ and the Jaccard index.^48^ For each image pair, we visualised the registration results corresponding to the highest MI value, and the highest Jaccard index value, as well as the lowest cross entropy value. These outcomes were then evaluated by two co‐authors, a microscopist and a neuroscientist. Their assessments consistently showed that the MI metric provided the robust image registration results. As a result, we chose to proceed with the MI metric.

### 3.3 Seamless navigation of multiscale vCLEM images using NavROI

We developed NavROI for intuitive navigation of image registration results. NavROI empowers users to:

1. Visualise 3D relationships: Grasp the spatial context of ROIs within the entire block after LM imaging and registration.
2. Optimise block trimming: Minimise block size by accurately locating ROIs on the ultramicrotome camera view, maximising section count per grid slot.
3. Estimate cutting depth: Visually assess the proximity of the latest section to the ROI depth.
4. Trace ultrastructure: Seamlessly navigate between ROI FM and ROI TEM maps to explore intricate 3D details, such as axons.

Our method extends the capabilities of Fiji's Correlia plugin^49^ into 3D.

### 3.4 Analysis of cutting depth estimation

We observed a strong linear correlation between the estimated depths of sections and their corresponding feeding values. This linear relationship supports the validity of using image registration to estimate cutting depth. Deviations were noted in deeper regions, potentially due to the decreased contrast in TEM images. For these regions, we utilised Section TEM maps for image registration. The reduced contrast in these deeper Section TEM maps can introduce uncertainties in segmentation and shape determination.

Depth estimation based on image registration contains inherent uncertainty, arising from both the object's structural complexity and the imaging system's limitations. For cylindrical objects aligned with the depth axis, especially those with minimal thickness variation, accurate depth estimation is particularly challenging. In images captured with a long depth‐of‐focus objective lens, objects can appear stretched in the depth dimension within the image stack. Considering these factors, we estimate the depth uncertainty to be approximately 2 µm.

### 3.5 Comparison of the motorised ultramicrotome approach for targeted sectioning

The motorised ultramicrotome^50^ leverages X‐ray micro‐CT data to automatically section blocks to a specific target plane. This automation streamlines the ultramicrotomy process, albeit requiring access to X‐ray micro‐CT. In contrast, the integrated workflow eliminates this need. Cutting depths of sections were estimated using Section FMs and Section TEM maps; however, this strategy can be applied to sections without fluorescence by using completely Section TEM maps for depth estimation, making our workflow a viable option for targeted vCLEM.

One of the key barriers preventing democratisation of vCLEM technology by biologists despite its significant potential to yield valuable insights is the considerable amount of time required to integrate it into their research workflows. Recent advancements in multimodal image registration and image segmentation algorithms would make it easier to navigate targets across multiple modalities and spatial scales, particularly when applied to vCLEM navigation up to TEM tomography. Our integrated workflow significantly improves both of robustness and efficiency of ROI navigation for vCLEM analysis by combining these recent advancements and implementing a lot of pragmatic day‐to‐day experience.

## 4 MATERIALS AND METHODS

### 4.1 Immunolabelling

Viral injection and transcardial perfusion fixation were performed to male mice by following a recent article.^51^ Cortical regions containing infected neurons were incubated for 2‐h in a blocking buffer (10% goat serum, 5% BSA in high osmolarity PB). They were then incubated overnight with primary antibody against GFP at dilution of 1:1000 in antibody solution (5% goat serum, 1% BSA in high osmolarity PB). Following three 5 min washes in high osmolarity PB, slices were incubated for 1 h (RT) with nanobody at a dilution of 1:500 (FluoTag‐X4 anti‐RFP AZDye568; Nanotag Biotechnologies) and secondary antibodies (Anti‐Chicken 488, Invitrogen) at a dilution of 1:1000 in antibody solution and rinsed with PBS (4 times 10 min each). Finally, sections were incubated in 1:200 dilution of Lycopersicon Esculentum Lectin (LEL), DyLight649 (Vector Laboratories) and DAPI (1 µg/mL) in PBS for 30 min at RT.

### 4.2 Confocal microscopy

#### 4.2.1 Block FM, ROI FM and Surface DIC map

Resin embedded tissue blocks were imaged either a Nikon AX‐R NSPARC or a SoRA confocal microscope using a custom built imaging chamber. Low magnification reference stacks (Block FMs) were imaged using 20 X air objectives coupled with a DIC optics using the 405, 488, 543 and 650 nm laser lines and collected with the appropriate emission filters. Transmitted light images were acquired simultaneously using one of the laser beams (Surface DIC map). Higher resolution image stacks (ROI FMs) were obtained using a 60× oil immersion objective.

#### 4.2.2 Section FM

Ultrathin sections (200–300 nm) were either collected onto glass slides or collected in slot grids and placed onto 35 mm plastic petri dishes containing PBS. They were imaged using an inverted confocal Nikon AX‐R NSPARC.

### 4.3 EM sample preparation

EM sample preparation was performed by following a previous article:^18^ Samples were frozen with polyvinylpyrrolidone as a cryoprotectant, in an EM ICE high pressure freezer (Leica). Freeze substitution and resin embedding were performed in an automated AFS2 machine (Leica), using the freeze substitution processor unit. To facilitate the infiltration of Lowicryl HM20 (Polysciences Inc), the temperature was gradually raised to −25°C while increasing the resin concentration in acetone, and the samples were UV polymerised at −25°C through to +25°C.

### 4.4 Ultramicrotomy

Blocks were sectioned using a UC7 (Leica, Wetzlar, Germany). Section thickness was 200 nm. Feeding values was recorded for cutting each section.

### 4.5 TEM imaging

#### 4.5.1 Section TEM map

The entire section was imaged using a TEM, JEM‐1400 Flash (Jeol, Tokyo, Japan) with acceleration voltage of 80 kV, with Limitless Panorama montaging software. Typical montage size was 400 × 400 µm^2^. The pixel size (*X*/*Y*) was 340 nm.

#### 4.5.2 ROI TEM map

The region round the ROI was imaged by TEM as above, except typical size was 70 × 100 µm^2^. The pixel size (*X*/*Y*) was 3.4 nm.

#### 4.5.3 TEM tomography

The regions round the ROI were imaged using a JEM‐1400 Flash (Jeol, Tokyo, Japan) TEM equipped with a 2040 dual axis holder (Fischione), operating at an accelerating voltage of 120 kV. Tilt series were acquired over ± 60° in 1° increments in two axes with 90° rotation using SerialEM.^9^ The typical size was 4 × 4 × 0.2 µm^3^ volume. Voxel size was 1.8 nm. The dual axis tomogram was reconstructed using weighted back projection in the Etomo package of IMOD^52^, ^53^ software with around 5 of the highest tilt angle images excluded.

### 4.6 Image preprocessing for registration

Prior to applying for SegReg to multiscale images, pixel resolutions of these images were matched using Fiji. All the image processing was performed using the NVIDIA A5000 graphics processing unit.

For Section FMs, raw image stacks were MIP projected after drift in *Z* direction was corrected using Correct 3D Drift plugin.^54^

For nonlinear image registration between adjacent ROI TEM maps, approximately 30 corresponding points were manually identified and registered using bUnwarpJ plugin.^55^

### 4.7 Segmentation

#### 4.7.1 Blood vessels

Image binarisation was used in LM images. The threshold for binarisation was determined using the maximum entropy method.^56^ When a threshold value is set, pixels with intensity values below the threshold are classified as background, while those above the threshold are classified as the object. The entropy is calculated separately for each class, and the two values are summed. As the threshold value is adjusted, the sum of the entropies changes. The maximum entropy method identifies the threshold value that results in the highest sum of entropies, thereby optimising the classification of pixels. We found empirically that the maximum entropy thresholding method consistently performs well for our fluorescent LM images.

#### 4.7.2 Cell nuclei

Cell nuclei segmentation algorithms have been actively proposed for both LM and TEM images. The SAMJ plugin^57^ in Fiji^58^ was used. The SAM plugin^59^ in napari can also be used. Users identify cell nuclei while viewing LM images and annotate boxes around the identified nuclei. Using the annotated boxes as a prompt, the algorithm segments the cell nuclei.

#### 4.7.3 Neurons

Image binarisation was used for LM images. The threshold for binarisation was determined using the maximum entropy method,^56^ since we found empirically that this method consistently performs well for our fluorescent LM images. Manual segmentation was performed for TEM images.

#### 4.7.4 Synapses

Image binarisation was used for LM images. The threshold for binarisation was determined using the maximum entropy method,^56^ since we found empirically that this method consistently performs well for our fluorescent LM images. Manual segmentation of presynaptic boutons was performed for TEM tomograms.

## Supporting information



Supporting Information

## Data Availability

The datasets supporting the findings of this study are openly accessible and have been deposited in public repositories. Light microscopy data (Block FM, ROI FM, Surface DIC map, Section FM) are available from the BioImage Archive under accession number S‐BIAD2077 (https://www.ebi.ac.uk/biostudies/bioimages/studies/S‐BIAD2077). TEM data can be found in EMPIAR under accession number EMPIAR‐12850 (https://www.ebi.ac.uk/empiar/EMPIAR‐12850/). Codes for SegReg and NavROI are available on GitHub (https://github.com/jburrone/Konishi_Burrone_2025) under MIT license.
