# Supplementary material for: A workflow for semi‐automated volume correlative light microscopy and transmission electron tomography
Source: J Microsc. 2025 Jun 30;300(1):94–107. doi: 10.1111/jmi.13436 (PMC12459417; doi:10.1111/jmi.13436)
Supplement: Supplementary file 1 — Supporting Information [file JMI-300-94-s001.docx]

**Supplementary Information**

**A workflow for semi-automated volume correlative light microscopy and transmission electron tomography**

Kohki Konishi^1,2,3*^, Guilherme Neves^3^, Matthew Russell^4^, Masafumi Mimura^2^, Juan Burrone^3*#^, Roland Fleck^4*#^

^1^ Nikon U.K., Branch of Nikon Europe B.V., Surrey, United Kingdom

^2^ Mathematical Science Research Laboratory, Nikon Corporation, Tokyo, Japan

^3^ Centre for Developmental Neurobiology, Institute of Psychiatry, Psychology & Neuroscience, King's College London, London, United Kingdom

^4^ Centre for Ultrastructural Imaging, King's College London, London, United Kingdom

* Corresponding authors: [kohki.konishi@nikon.com](mailto:kohki.konishi@nikon.com), [juan.burrone@kcl.ac.uk](mailto:juan.burrone@kcl.ac.uk), [roland.fleck@kcl.ac.uk](mailto:roland.fleck@kcl.ac.uk)

# These authors contributed equally to this work.


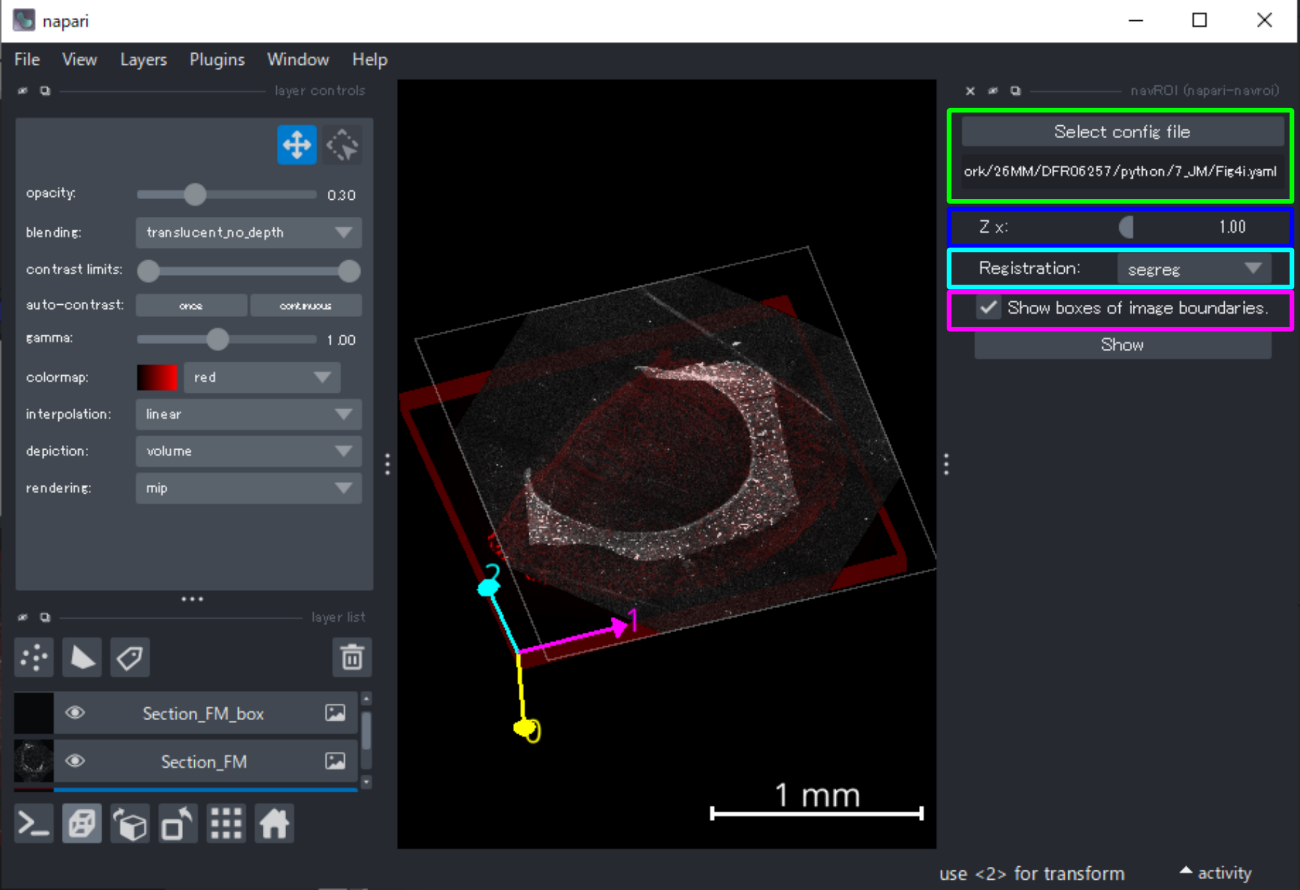


**Supplementary Figure 1: Interface of NavROI plugin.** This plugin visualizes the image registration results across multimodal and multiscale image stacks. A user first selects a configuration file (green rectangle). Then the user designates the Z magnification factor compared to the X and Y (blue rectangle), an image registration method (cyan rectangle, “segreg” or “landmark”), and select/unselect whether image boundaries are displayed (magenta rectangle).
